# Predictors of Postpartum Depression among Italian Women: A Longitudinal Study

**DOI:** 10.3390/ijerph19031553

**Published:** 2022-01-29

**Authors:** Sara Molgora, Emanuela Saita, Maurizio Barbieri Carones, Enrico Ferrazzi, Federica Facchin

**Affiliations:** 1 Department of Psychology, Università Cattolica del Sacro Cuore, 20123 Milan, Italy; emanuela.saita@unicatt.it (E.S.); federica.facchin@unicatt.it (F.F.); 2 Fondazione IRCCS Cà Granda, Ospedale Maggiore Policlinico, 20122 Milan, Italy; m.barbiericarones@gmail.com (M.B.C.); enrico.ferrazzi@unimi.it (E.F.); 3 Department of Clinical Science and Community Health, Università degli Studi di Milano, 20122 Milan, Italy

**Keywords:** postpartum depression, predictors, longitudinal study, anxiety, childbirth experience

## Abstract

Introduction: Postpartum depression is commonly experienced by mothers worldwide and is associated with anxiety disorders, parenting stress, and other forms of distress, which may lead to a complex illness condition. Several studies have investigated the risk factors for this disorder, including biological and socio-demographic variables, medical and obstetric factors, and psychological and relational dimensions. The present study aimed to describe the psychological status of mothers up to 12 months postpartum, and to investigate the predictors of depressive symptoms at 12 months postpartum, considering obstetric factors along with psychological and relational variables. Methods: A sample of 137 women completed a questionnaire composed of a sheet on anamnestic and obstetric information and the following scales: Wijma Delivery Experience Questionnaire; State-Trait Anxiety Inventory; Edinburgh Postnatal Depression Scale; Parenting Stress Index (Short Form); Dyadic Adjustment Scale; and Multidimensional Scale of Perceived Social Support. Data were collected at four assessment times: 2–3 days, 3 months, 6 months, and 12 months postpartum. Results: Findings showed that the highest percentage of women with clinically significant symptoms of anxiety (state and trait) and depression was found at 12 months postpartum, which indicated that this was the most critical time. The quality of childbirth experience and trait anxiety at three months postpartum emerged as significant predictors of postpartum depression at 12 months. Conclusion: Our findings highlight the importance of providing stable programs (such as educational programs) to mothers in the first year postpartum. Furthermore, because the quality of the childbirth experience is one of the most important predictors of PPD at 12 months postpartum, effort should be made by healthcare professionals to guarantee a positive experience to all women to reduce possible negative long-term consequences of this experience.

## 1. Introduction

The birth of a child significantly impacts a women’s psychological well-being and may lead to several forms of diseases, ranging from baby blues to more severe conditions such as anxiety disorders, depression, puerperal psychosis, and post-traumatic stress disorders [1,2,3,4,5]. 

Postpartum depression (PPD) represents an important clinical problem because it is frequently experienced by mothers worldwide, as demonstrated in several recent meta-analyses (e.g., [6,7,8,9]). PPD compromises women’s psychological health and, in some instances, may lead to suicidal behaviours [10]; it can also impair the relationship with the partner and the baby, with negative consequences on the child’s development [11]. 

PPD can be associated with anxiety disorders and parenting stress, deriving from a poor perceived ability to cope with the multiple challenges related to the new parental role [12], especially in first-time mothers [1,13,14,15,16]. PPD is also predicted by a variety of risk factors, such as biological (e.g., levels of specific hormones) and/or sociodemographic (e.g., socio-economic status) factors [17,18], medical and obstetrics variables related to pregnancy, labor and delivery [19], and psychological variables [20]. For example, complications during pregnancy (e.g., gestational diabetes, preeclampsia, thyroid autoimmunity) are associated with higher levels of PPD [19,21,22,23], which are also predicted by either elective or emergency caesarean section [24,25,26], although overall research findings are inconsistent [27]. In addition, mothers of preterm infants are more likely to develop PPD [28]. Most studies have investigated the predictive role of these factors on PPD in the first months postpartum, whereas few studies have examined their long-term impact on PPD. For this reason, PPD and its risk factors 1 year after giving birth remain unclear [20].

Furthermore, there is evidence of a significant association between PPD and previous anxiety or depression disorders, breastfeeding self-efficacy, and low maternal self-efficacy [29,30,31,32,33,34]. The subjective experience of childbirth can also affect women’s postpartum psychological well-being, with a negative experience being associated with higher levels of PPD [35,36,37]. 

Regarding relational variables, poor couple relationships and low social support by the formal and the informal network are important risk factors for PPD [29,33,38,39]. Furthermore, maternal violence experiences were significantly associated with an increased risk of developing PPD [38,40].

Moreover, specific contextual variables such as stressful life events are associated with a high prevalence of PPD [41]. The current COVID-19 pandemic can be considered as an additional stressful condition that may affect mothers’ psychological well-being [42]. In this regard, an increasing number of studies investigating the psychological impact of the pandemic showed higher levels of anxiety and depressive symptoms among postpartum women during the COVID-19 pandemic compared to similar cohorts assessed before the pandemic [43,44,45,46]. These findings can be explained considering both mothers’ concerns about the risk of coronavirus for themselves and the baby, and the reduced support received in this period [42]. 

Many studies on PPD are cross-sectional and focus on the prevalence and the correlates of PPD; prospective studies mainly consider a limited time frame, without including long-term effects of childbirth or the evolution of PPD symptoms over time [47,48]. In this study, we considered a longer postpartum time frame (1 year). Moreover, the longitudinal design allows for a more in-depth understanding of the effects of becoming a mother on women’s psychological health, considering childbirth as a complex event [48].

The aims of this study were to: (1) describe the psychological (depression, anxiety, parenting stress) and relational (couple adjustment, perceived social support) status of mothers up to 12 months postpartum; and (2) identify the main predictors of PPD at 12 months postpartum, considering obstetrics factors, and psychological and relational variables. Based on previous studies, we expected that PPD would be predicted by either individual or relational variables, with higher levels of depression associated with lower levels of social support and couple adjustment, and a more negative experience of childbirth. Furthermore, we expected that PPD at 12 months postpartum would be predicted by previous psychological distress, i.e., PPD, anxiety, and parenting stress at 3 months postpartum. 

## 2. Methods

### 2.1. Procedures and Participants

This was a longitudinal study that comprised 137 Italian postpartum women, recruited between October 2019 and March 2021 in a public hospital located in Northern Italy. Inclusion criteria were being a postpartum women aged ≥18 years and fluent in Italian. Eligible participants received complete information about all the aspects of the research by a member of the research team during postpartum hospitalization. All the women who accepted our invitation to participate in the study provided written informed consent. Ethical approval was received by the Institutional Review Board (approval number 922_2019bis; approval date: 9 October 2019). 

Data collection involved four assessment times: 2–3 days after-delivery (Time 1), after 3 months (Time 2), 6 months (Time 3), and 12 months (Time 4). At Time 1, women completed the questionnaires at the hospital, whereas at Time 2, 3, and 4 the questionnaires were completed on the Qualtrics platform following a reminder by email. A total of 323 eligible participants were initially identified. Of these, 2 did not meet the inclusion criteria (age < 18 years) and were excluded from the study. Therefore, 321 women completed the questionnaires at Time 1. Overall, incomplete information (>80% of missing data) was reported by 184 participants, whose data were not used in the final statistical analyses. In total, the questionnaires were returned at all of the four assessment times by 137 participants. 

### 2.2. Measures

At Time 1, women completed a sheet focused on sociodemographic (age, education, employment status, parity) and obstetric information (gestational age, labor induction, mode of delivery, use of epidural analgesia, episiotomy). Women also provided information regarding previous psychological disorders (e.g., depression, anxiety, eating disorders, alcoholism, drug addiction), distressing experiences before pregnancy (such as previous miscarriages), conception (spontaneous or using assisted reproductive technology), and type of pregnancy (including complications during pregnancy and threat of miscarriage). We subsequently collected information about mode of feeding (at Time 2; i.e., “How do you feed your child?”, 4 possible responses: exclusive breastfeeding, exclusive artificial milk, mixed, other). Moreover, although the questionnaire was not specifically aimed at investigating the impact of COVID-19 on mothers’ psychological health, we included two questions related to the pandemic—(1) To what extent do you think your health and that of your child are threatened by the pandemic? (2) To what extent has the pandemic impacted on your life?—with responses scored on a 0 (Not at all) to 5 (Extremely) Likert scale. Because Time 1 occurred before the COVID-19 outbreak, these two questions were asked at Time 2, 3, and 4.

At Time 1 and 2, women completed the Wijma Delivery Experience Questionnaire-WDEQ(B) [49,50]. The Italian-validated version evaluates the childbirth experience through 14 items on a 6-point Likert scale; the total score ranges from 0 to 70, with a higher score indicating a more negative experience. Internal consistency was good (Cronbach’s alpha = 0.86) at Time 1 and very good (Cronbach’s alpha = 0.91) at Time 2. We considered a score of 39 as the cut-off value to identify cases of severe fear of childbirth [51]. 

At Time 2, Time 3 and Time 4, women also completed the following instruments:

- Edinburgh Postnatal Depression Scale—EPDS [52,53]. This instrument is composed of 10 items on a 4-point Likert scale, with a total score ranging from 0 to 30: the higher the score, the higher the depressive symptoms. Internal consistency was good, ranging from 0.84 at Time 2 to 0.88 at Time 4. According to Benvenuti and colleagues [53], a cut-off value of 9 or higher was used to distinguish clinical depression, whereas according to Gibson and colleagues [54] the cut-off value is fixed at 12 or higher.
- State-Trait Anxiety Inventory–STAI, Y form [55,56]. This instrument is composed of 40 items (20 items for trait anxiety and 20 items for state anxiety) on a 4-point Likert scale, with a total score of 20–80: the higher the score, the higher the anxiety symptoms. Internal consistency was very good for both the state (Cronbach’s alpha = 0.94 at Time 2, and 0.95 at Time 3 and Time 4) and the trait (Cronbach’s alpha = 0.90 at Time 2 and Time 4, and 0.88 at Time 3) subscales. Based on previous studies on similar cohorts, a cut-off score of 40 or higher was used to identify both state and trait clinical anxiety [15,57].
- Parenting Stress Index—PSI [12,58]. This scale is composed of 36 items on a 5-point Likert scale, with a total score ranging from 36 to 180: the higher the score, the higher the perceived level of global parenting stress. Internal consistency was very good, ranging from 0.92 at Time 3 to 0.95 at Time 2. We considered a score of 90 as the cut-off value to identify high levels of parenting stress [58].
- Dyadic Adjustment Scale—DAS [59,60]. This scale is composed of 32 items, of which 31 are related to couple adjustment, and one item refers to the overall perceived happiness with the relationship. The total score ranges from 0 to 151: the higher the score, the higher the couple adjustment. Internal consistency was very good (Cronbach’s alpha ranging from 0.94 at Time 2 to 0.96 at Time 4).
- Multidimensional Scale of Perceived Social Support—MSPSS [61,62]. This instrument is composed of 12 items, with a 12–80 total score range, and measures the perception of social support from three different sources (family, friends, and significant others); the higher the score, the higher the perceived social support. Internal consistency was very good (Cronbach’s alpha = 0.93 at all times).

### 2.3. Statistical Analyses

Statistical analyses were performed using SPSS software, version 27 (IBM, New York, NY, USA). Descriptive statistics were computed to summarize the participant characteristics. Means and standard deviations (SDs) were reported for continuous variables (WDEQ(B), EPDS, STAI, PSI, DAS, MSPSS), and frequencies and percentages for categorical variables. Continuous psychological health outcomes (data collected using the WDEQ(B), the EPDS, the STAI, and the PSI) were also dichotomized using the cut-offs of each scale, to establish the percentage of clinical subsamples for each scale. Normality of distribution was verified at all the assessment times, considering skewness and kurtosis. Values ranging between −2 and +2 indicated that the data distribution was approximately normal [63]. Only the social support (MSPSS) at T4 and couple adjustment (DAS) at T3 and T4 did not have a normal distribution and were excluded from the analyses. 

First, we used independent samples t-test and chi-squared test (as appropriate) to compare the women who abandoned the study after Time 1 with those who returned the questionnaires at all the assessment times. The effect of time on the psychological and relational variables was examined using repeated measures ANOVA. To identify the predictors of women’s postpartum depression at Time 4, Pearson correlations were performed for continuous variables, whereas univariate ANOVAs were performed for categorial independent variables. Those factors that were significantly related to PPD were subsequently included in a multivariable regression model. All the categorical predictors entered into the regression models were dichotomous (i.e., previous stressful event) or were dichotomized and recoded as dummy variables [64]. For instance, the pandemic-related perceived threat was recoded as low threat (scores between 0 and 3) or high threat (scores from 4 to 5). Statistical significance was set at *p* < 0.05.

## 3. Results

### 3.1. Participant Characteristics

Women’s age was 34.91 (SD = 4.0; range= 24–44). The 137 women who agreed to participate in the study at all assessment times were more likely to be first-time mothers (χ^2^(1,137) = 4.75; *p* = 0.029) and to have a higher level of education (χ^2^(5,137) = 21.07; *p* = 0.001) than those who abandoned the study after Time 1. As regards the other socio-demographic, obstetric, and psychological variables, no significant differences emerged between the two groups. The majority of the 137 final participants had an academic degree (55.5%), was employed (67.9%), and was married (63.4%) or cohabiting (36.6%). The mean length of the couple relationship was 8.21 years (SD = 4.5). 

Most participants (67.9%) were primiparae, conceived spontaneously (83.9%), and had a vaginal birth (59.9%), vs. 38% of women who had a caesarean section (of which 40.4% had a planned caesarean section, 42.3% had an emergency caesarean section, and 17.3% had an elective caesarean section) and 2.1% who had operative delivery. As regards mode of feeding, 60.2% reported exclusive breastfeeding, 17.2% used artificial milk, and 20.3% used both breast and artificial milk (mixed). 

A minority of women (27.2% at T2, 17.3% at T3, and 24.1% at T4) reported a high perceived threat for their own health and that of their child in relation to COVID-19. Considering the impact of the pandemic on women’s life, the majority of participants reported a perceived high impact (68.3% at T2, 44.8% at T3, and 61.4% at T4). Further sociodemographic and obstetric information is reported in Table 1. 

### 3.2. Women’s Psychological and Relational Status

Table 2 reports women’s scores for all the psychological variables (individual and relational) at all of the four assessment times. Repeated measures ANOVAs showed no differences among the times of assessment, except for trait anxiety, which was significantly higher at Time 4 than the other times (F = 3.9; *p* = 0.022).

Table 3 shows the percentages of mothers who reported clinically significant symptoms of anxiety and depression, parenting stress, and negative quality of childbirth experience, considering the cut-off scores of the scales. The highest percentage of women with clinically significant symptoms of anxiety (state and trait) and depression was found at Time 4, which indicated that this was the most critical time. Furthermore, 7% and 17% of women—considering a cut-off score of 9 and 12, respectively—reported clinically significant depressive symptoms at all of the three assessment points.

The Pearson’s correlations reported in Table 4 showed that PPD at 12 months (Time 4) postpartum was positively correlated with the quality of childbirth experience at three months (Time 2), and with anxiety and depression at all assessment times. Conversely, PPD was negatively associated with social support and couple adjustment.

The ANOVAs conducted to detect group differences, based on sociodemographic (parity) and obstetric (type of delivery, mode of conception, complications during pregnancy, epidural analgesia, episiotomy, induction, weeks of gestation) factors in depressive symptoms at 12 months postpartum (Time 4) showed no statistically significant results. In addition, mode of feeding did not have an impact on PPD at 12 months postpartum. 

Women who had experienced one or more stressful events (e.g., economic problems, work problems, own illness, or illness of a significant person, etc.) during pregnancy or in the postpartum (Time 1) reported greater depressive symptoms at 12 months postpartum (Time 4; F = 8.53; *p* = 0.004). Furthermore, women who reported a high perceived threat related to COVID-19 at three months postpartum (Time 2) showed greater depressive symptoms at 12 months postpartum (Time 4; F = 4.29; *p* = 0.043). Finally, the quality of childbirth experience at three months (Time 2) had a significant impact on PPD, with women reporting a critical or even traumatic experience (cut-off score above 39) showing higher levels of depressive symptoms at 12 months postpartum (Time 4; F = 12.64; *p* = 0.001). In particular, the chi-squared test showed that women who had a negative experience of childbirth at three months postpartum (Time 2) were more likely to report clinically significant depressive symptoms at 12 months postpartum (Time 4; χ^2^(1,111)=13.60; *p* = 0.000).

We subsequently performed a linear regression, including previous stressful event (assessed at Time 1), perception of COVID-19 threat at three months, quality of childbirth experience at three months, anxiety (state and trait), depression and parenting stress at three months, couple adjustment, and social support at three months as predictors of PPD at 12 months. The findings of this analysis are reported in Table 5 and showed statistically significant results for two predictors: the quality of childbirth experience and trait anxiety. Conversely, the other variables did not significantly predict postpartum depression at one year. The model (F (_8,36_) = 4.42; *p* < 0.001) explains 38% of the total variance of the dependent variable (R_2_ = 0.38).

## 4. Discussion

Because the birth of a child represents a critical and potentially stressful experience with possible negative consequences on women’s mental health [1,2,3,4,5], the primary aim of this study was to describe the psychological status of mothers up to 12 months postpartum. Indeed, this longitudinal framework, which is broader than that of other longitudinal studies or cross-sectional studies, allows more in-depth analysis of the psychological impact of transitioning to parenthood, also considering that women’s psychological status in the postpartum period can change over time.

Our findings showed that trait anxiety was significantly higher at 12 months postpartum; furthermore, the highest percentage of women with clinically significant symptoms of state and trait anxiety and depression was found at 12 months postpartum. These results interestingly confirmed the findings of another recent study [65], in which the highest levels of depression were detected at 9–12 months postpartum, and suggest that approximately one year after birth represents one of the most critical and challenging time windows in the postpartum period (this is a useful information, also considering the paucity of research investigating women’s psychological health 12 months after childbirth). In another study on fathers’ trajectories of postpartum depression, the men participants reported the highest percentage of depressive symptoms at one year postpartum [48]. We can speculate that this is a critical time because in Italy it usually coincides with the end of maternity leave and the return to work, with the concomitant admission of the child to the kindergarten. For these reasons, it may represent a complex time for women who have to manage both work and family commitments. These findings also underline the importance of longitudinal studies to examine the psychological wellbeing of new parents over time, which may also highlight the possible long-term consequences of the transition to parenthood. 

Furthermore, our findings showed higher percentages of women with clinically significant psychological symptoms (considering all of the four assessment times) compared with those reported in the pre-pandemic literature on similar cohorts, which suggests that in our sample the experience of motherhood was also shaped by pandemic-related factors [42], especially the pandemic-related perceived threat. The overall estimated prevalence of anxiety disorders/symptoms in this population was around 10–15% before the pandemic [66]; in our sample, the percentage of women above the clinical cut-off score was more than double at 3 and 6 months postpartum, and approximately more than triple at 12 months postpartum. These percentages are in line with those of a previous study carried out during the first lockdown of the pandemic [42]. At the same time, in our sample, the percentage of women with clinically significant symptoms of depression was higher than those reported in the pre-pandemic literature [67,68,69]. For up to the 17% of the participants who reported clinically significant symptoms of depression, the levels of depression were clinically significant at all of the three assessment points, which indicated a stable but critical situation. These findings confirm those of previous studies that identified a high-risk trajectory, with relevant depressive symptoms at all assessment points [70,71,72], and provide useful information for clinical intervention, underlining the importance of continuous support for postpartum women. However, the highest percentage of stable high-risk women found in this study highlights the significant impact of the pandemic on mothers’ well-being, which further underlines the importance of offering supportive interventions not only immediately after childbirth, but also throughout the following year. Regarding the childbirth experience, approximately one-third of women reported a very negative experience, emphasizing how the experience of childbirth during the pandemic was negative for many women, as highlighted in a previous study [73]. 

Finally, the presence of multiple correlations between the psychological and the relational variables included in the study indicates a complex condition of psychological distress that cannot be reduced to depressive symptoms alone. In this scenario, relational variables can play a protective role, as has been well documented in both the pre-pandemic and pandemic literature [44,74]. 

Regarding the main predictors of PPD at 12 months postpartum (the second aim of our study), our findings showed significant associations with the quality of childbirth experience and trait anxiety. This result only partially confirms our hypothesis. As expected, PPD was predicted by the quality of childbirth experience and anxiety at three months, which is in line with findings of previous studies. Specifically, several studies found an association between a negative subjective experience of childbirth and maternal depression [36,37,75,76,77,78,79], which highlights the importance of improving the quality of the childbirth experience to reduce its possible negative consequences on women’s well-being, and on the relationship with the baby and the baby’s development [80,81]. 

Furthermore, the significant predictive role of trait anxiety on PPD at 12 months indicates that “structural” rather than situational factors have an impact on depressive symptoms. This result confirms those from previous studies that underlined the continuity of psychological distress across the transition to parenthood [82]. Surprisingly, in our study, PPD at previous assessment times did not affect PPD at 12 months. Taken together, these findings may suggest that an initial condition of anxiety in mothers, if untreated, may lead to long-term negative consequences including PPD. 

On the contrary, neither couple adjustment nor social support were found to be predictive of PPD, although they were negatively correlated with PPD. Therefore, although relational variables can have a protective role in mothers’ psychological adjustment, PPD is directly predicted by individual variables related to psychological dimensions, and especially to the quality of childbirth experience.

The current study has some limitations. First, more than half of the participants abandoned the study. Second, two scales (DAS and MSPSS) did not have a normal distribution and for this reason they were not included in all the analyses conducted. Third, mothers’ well-being was investigated using only self-report instruments, exposed to social desirability bias. Future studies could investigate mothers’ psychological health using qualitative designs based on in-depth interviews, to better understand the subjective experience of the transition to parenthood. Finally, although this study was not originally focused on motherhood experience during the pandemic, this unexpected event inevitably had an impact on our research, so that some questions related to COVID-19 needed to be included.

Despite these limitations, the longitudinal design of our study allows a longer period of time to be covered compared with other studies, and can provide useful information to plan specific support interventions for postpartum women. For instance, although antenatal classes are routinely offered to Italian expectant mothers, it may be useful to provide stable programs (such as educational programs) to mothers in the first year postpartum. Because the quality of the childbirth experience is the most important predictor of PPD, effort should be made by healthcare professionals to guarantee a positive experience to all women. 

## 5. Conclusions

Investigating women’s psychological status in the postpartum period is essential to understand how we can support women through targeted interventions based on their specific needs. In this regard, our findings may usefully contribute to research and clinical practice by showing that the quality of the childbirth experience has long-term effects on women’s psychological well-being. The fact that the whole first year represents a window of vulnerability, rather than only the first months after giving birth, should be considered by healthcare professionals in clinical practice with mothers. 

## Figures and Tables

**Table 1:** Participants’ sociodemographic and obstetric information.

	N (137)	%
SOCIODEMOGRAPHIC INFORMATION *Level of education*		
Professional licensing course	2	1.4
High school	40	29.2
Degree/graduate specialization	76	55.5
PhD/post-graduate specialization	19	13.9
*Occupational status*		
Self-employed	17	12.4
Employed	93	67.9
Unemployed	9	6.6
Housewife	7	5.1
Student	2	1.4
Other	9	6.6
*Distressing experience*		
No stressful event	104	75.9
At least one stressful event (economic problems, work-related problems, health problems, bereavement, etc.)	33	24.1
*Previous psychological disorders*		
No	97	70.8
Yes	40	29.2
OBSTETRIC INFORMATION		
*Type of pregnancy*		
Single	131	95.6
Twin	6	4.4
*Previous miscarriage*		
No	103	75.2
Yes	34	24.8
*Complications during pregnancy*		
No complication	79	57.7
At least one complication (threatened miscarriage, detached placenta, hypertension, gestational diabetes, etc.)	58	42.3
*Gestational age*		
*≤37*	24	20.7
*≥38*	38	79.3
*Rupture of membranes*		
No	109	80.1
Yes	27	19.9
*Induction*		
No	72	52.9
Yes	64	47.1
*Epidural*		
No	41	30.1
Yes	95	69.9
*Episiotomy*		
No	100	74.6
Yes	34	25.4

**Table 2:** Repeated measures ANOVA: differences among times of assessment for the psychological and relational variables.

	Time 1	Time 2	Time 3	Time 4	
	M(SD)	M(SD)	M(SD)	M(SD)	*p*
WDEQ-B	26.2 (12.7)	27.7 (12.1)	/	/	0.225
EPDS	/	7.7 (5.0)	7.4 (5.0)	7.8 (5.4)	0.708
STAI-State	/	38.0 (10.4)	38.4 (10.7)	40.3 (10.4)	0.171
STAI-Trait	/	37.8 (9.1)	38.2 (8.3)	39.5 (9.2)	0.022
PSI	/	67.2 (19.6)	65.7 (16.6)	66.9 (17.6)	0.600
MSPSS	/	68.4 (12.6)	68.5 (12.0)	/	0.131
DAS	/	118.0 (18.6)	/	/	/

**Table 3:** Percentage of women above the clinical cut-off for psychological variables across times.

Scale (Cut-Off Core)	Time 1	Time 2	Time 3	Time 4
WDEQ-B (39)	29.7	25.2	/	/
EPDS (12)	/	20.3	21.3	21.9
EPDS (9)		40.6	36.0	40.9
STAI-State (40)	/	33.6	35.3	46.0
STAI-Trait (40)	/	35.4	39.0	44.5
PSI (90)	/	10.2	6.1	9.5

**Table 4:** Pearson’s correlation matrix among psychological and relational variables at all times of assessment.

	WDEQ(B)_t1	WDEQ(B)_t2	STAI_S_t2	STAI_T_t2	EPDS_t2	PSI_t2	DAS_t2	MSPSS_t2	STAI_S_t3	STAI_T_t3	EPDS_t3	PSI_t3	MSPSS_t3	STAI_S_t4	STAI_T_t4	EPDS_t4	PSI_t4
WDEQ(B)_t1		0.77 **	0.18	0.24 *	0.20	0.19	−0.09	−0.23 *	0.09	0.13	0.08	0.11	−0.21 *	0.16	0.26 **	0.18	0.24 *
WDEQ(B)_t2			0.39 **	0.36 **	0.40 **	0.37 **	−0.18	−0.37 **	0.25 **	0.29 **	0.25 **	0.31 **	−0.32 **	0.31 **	0.34 **	0.35 **	0.37 **
STAI_S_t2				0.78 **	0.76 **	0.58 **	−0.51 **	−0.49 **	0.50 **	0.53 **	0.53 **	0.48 **	−0.35 **	0.56 **	0.59 **	0.57 **	0.48 **
STAI_T_t2					0.68 **	0.61 **	−0.61 **	−0.47 **	0.59 **	0.76 **	0.60 **	0.48 **	−0.40 **	0.53 **	0.71 **	0.58 **	0.40 **
EPDS_t2						0.58 **	−0.37 **	−0.43 **	0.40 **	0.48 **	0.59 **	0.52 **	−0.33 **	0.44 **	0.52 **	0.54 **	0.54 **
PSI_t2							−0.49 **	−0.42 **	0.40 **	0.41 **	0.42 **	0.72 **	−0.34 **	0.35 **	0.36 **	0.38 **	−0.26 **
DAS_t2								0.35 **	−0.37 **	−0.49 **	−0.36 **	−0.41 **	0.29 **	−0.37 **	−0.47 **	−38 **	−0.29 **
MSPSS_t2									−0.16	−0.27 **	−0.13	−0.34 **	0.63 **	−0.16 *	−0.26 **	−25 **	−0.29 **
STAI_S_t3										0.78 **	0.53 **	0.50 **	−0.33 **	0.56 **	0.60 **	0.57 **	0.48 **
STAI_T_t3											0.72 **	0.49 **	−0.39 **	0.61 **	0.75 **	0.56 **	0.52 **
EPDS_t3												0.48 **	−0.19 *	0.51 **	0.53 **	0.57 **	0.46 **
PSI_t3													−0.40 **	0.40 **	0.40 **	0.36 **	0.70 **
MSPSS_t3														−0.23 **	−0.35 **	−19 *	−0.36 **
STAI_S_t4															0.79 **	0.77 *	0.55 **
STAI_T_t4																0.69 **	0.57 **
EPDS_t4																	0.50 **
PSI_t4																	

* *p* < 0.01, ** *p* < 0.001.

**Table 5:** Multiple linear regression: Effect of previous stressful events, WDEQ(B), STAI (state and trait), PSI, MSPSS, DAS and perception of threat related to COVID-19 at 3 months on EPDS at 12 months.

Predictors	*b*	SE *b*	β	*t*	*p*
Stressful event_t1	0.191	1.707	0.014	0.112	0.912
WDEQ(B)_t1	0.172	0.064	0.387	2.705	0.010
STAI_S_t1	0.024	0.107	0.053	0.228	0.821
STAI_T_t1	0.341	0.121	0.643	2.822	0.008
PSI_t1	−0.039	0.046	−0.164	−0.849	0.401
MSPSS_t1	0.114	0.065	0.289	1.759	0.087
DAS_t1	−0.036	0.059	−0.110	−0.606	0.548
Covid_Threat_t1	0.253	1.612	0.020	0.157	0.876

## Data Availability

Data are available on request contacting the authors.
